# Synergistic Anticancer Effects of Fermented Noni Extract Combined with 5-Fluorouracil, Doxorubicin, and Vincristine on A549, MCF-7, and SH-SY5Y Cell Models

**DOI:** 10.3390/cimb47120993

**Published:** 2025-11-27

**Authors:** June-Seok Lim, Ji-Hyun Im, Xiaolu Fu, Min-Hye Kim, Yeon-Seok Seong, Jae-Yeon Lee, Eun Young Park, Do sang Lee, Im-Joung La, Ok-Hwan Lee

**Affiliations:** 1 Department of Food Biotechnology and Environmental Science, Kangwon National University, Chuncheon 24341, Republic of Korea; 2 NSTBIO Co., Ltd., Incheon 21984, Republic of Korea; 3 Atomy R&D Center, Gongju 32511, Republic of Korea

**Keywords:** *Morinda citrifolia* L., fermented noni extract, combination index, synergistic anticancer effect, apoptosis

## Abstract

Although chemotherapy is a primary approach for cancer treatment, its efficacy is often compromised by the development of drug resistance and occurrence of serious side effects. To improve chemotherapy outcomes, this study aimed to evaluate the synergistic anticancer effects of fermented noni extract (FN) in combination with 5-fluorouracil (5-FU), doxorubicin (DOX), or vincristine (VIN) in A549, MCF-7, and SH-SY5Y cancer cell lines. Cells were treated with FN, anticancer drugs, or their combinations. Cell viability was assessed using XTT assays, and synergism was evaluated by calculating combination index (CI) values. Scratch wound and colony formation assays were conducted to assess cell migration and proliferation. Western blotting was used to analyze the expression of apoptosis-related proteins. Combination treatments significantly enhanced cytotoxicity (CI < 1), inhibited migration and colony formation, and induced intrinsic apoptosis. Expression ratios of BAX/BCL-2, cleaved caspase-9/caspase-9, and cleaved caspase-3/caspase-3 notably increased. These findings suggest that FN enhances the efficacy of chemotherapy drugs by promoting intrinsic apoptotic pathways and may serve as a potential natural adjuvant in cancer therapy.

## 1. Introduction

Noni fruit (*Morinda citrifolia* L.), a perennial species belonging to the *Rubiaceae* family, is native to the South Pacific Islands, including Indonesia, Tahiti, and Australia, and is commonly found in the tropical regions of China, including Hainan, Xisha, and Taiwan [1]. Traditionally used as an edible medicinal plant, noni is now marketed globally as a dietary supplement [2]. In traditional Polynesian and Pacific Island medicine, noni has been used to treat various ailments, including infections, inflammation, pain, and tumor-like conditions. Specifically, ethnomedical reports describe its use in managing swelling, abnormal growths, and symptoms resembling cancer [3,4]. These traditional applications support modern investigations into their potential anticancer effects. Various forms of noni products are available, including fermented juice, fresh juice, fruit powder, and fruit tea, among which fermented noni juice is the most widely consumed owing to its reported superior health benefits [5]. Noni fruits are rich in bioactive compounds, with more than 200 phytochemicals being identified to date [6]. These compounds exhibit a broad range of pharmacological activities, including anticancer, antidiabetic, antiobesity, antibacterial, antiviral, and anti-inflammatory effects [7]. Among them, the anticancer properties have been garnering considerable research interest in noni fruit extracts and phytochemicals [8]. Noni fruit can inhibit cancer metastasis, modulate major cancer-related signaling pathways [9], indirectly exert anticancer activity, and prolong life by activating the host immune system when combined with anticancer drugs, such as 5-fluorouracil (5-FU), doxorubicin (DOX), or vincristine (VIN) [10]. In addition,, ethanolic extracts of Thai noni juice enhanced the anticancer efficacy of 5-FU and reduced its toxicity in cholangiocarcinoma models, with no adverse effects observed in body weight, kidney, or spleen [11]. Furthermore, extensive toxicological evaluations have demonstrated that noni fruit juice is safe for human consumption. High-dose and long-term administration of noni juice produced no adverse effects in animal toxicity studies or human clinical trials, with normal liver, kidney, hematological, and biochemical parameters across all treatment groups [12]. However, the validity of the synergistic effect of this combined treatment and the mechanism of direct anticancer activity are unclear.

Combination therapy is widely adopted in clinical oncology owing to its improved treatment efficacy while minimizing the risk of drug resistance and reducing treatment-related adverse effects [13]. For instance, 5-FU primarily induces cytotoxicity by inhibiting thymidylate synthase, leading to DNA damage and activation of p53-mediated intrinsic apoptosis. It is commonly used in lung cancer therapy but is associated with hepatotoxicity, immunosuppression, diarrhea, and sensory neuropathy. DOX, a standard treatment for breast cancer, acts mainly by intercalating into DNA and inhibiting topoisomerase II, and it also generates reactive oxygen species that trigger mitochondrial apoptotic signaling. However, its clinical use is limited by cardiotoxicity and drug resistance. Similarly, VIN, which is effective against neuroblastoma, disrupts microtubule polymerization, causing mitotic arrest and activation of mitochondrial apoptosis. Nevertheless, its therapeutic potential is restricted by dose-limiting neurotoxicity [13,14,15]. Recent studies have highlighted the potential of natural compounds as combination partners to enhance anticancer efficacy and mitigate adverse effects [16].

Therefore, this study aimed to evaluate the synergistic anticancer effects of fermented noni extract (FN) in combination with standard chemotherapeutic agents (5-FU, DOX, and VIN) in lung (A549), breast (MCF-7), and neuroblastoma (SH-SY5Y) cancer cell lines. We investigated the effects of these combination treatments on cell viability, migration, proliferation, and apoptosis using XTT, cell migration, and colony formation assays, along with Western blotting analysis of apoptosis-related proteins. By identifying the mechanistic basis of the synergistic effects of FN, this study sought to provide new insights into the development of functional adjuvant strategies for improving chemotherapy outcomes with each anticancer drug while potentially reducing dose-dependent toxicity.

## 2. Materials and Methods

### 2.1. Chemicals and Standards

Trypsin-ethylenediaminetetraacetic acid, phosphate-buffered saline (PBS, pH 7.4), penicillin–streptomycin (P/S), fetal bovine serum (FBS), Ham’s F-12 Nutrient Mix (F-12), and Ham’s F-12K (F-12K) were purchased from Gibco (Carlsbad, CA, USA). Eagle’s Minimum Essential Medium (EMEM) was purchased from the American Type Culture Collection (ATCC, Manassas, VA, USA). Primary and secondary antibodies specific for p53 (1:1000), B-cell leukemia/lymphoma 2 protein (BCL-2) (1:1000), BCL-2-associated X protein (BAX) (1:1000), caspase-3 (1:1000), cleaved caspase-3 (1:1000), caspase-9 (1:1000), cleaved caspase-9 (1:1000), and β-actin (1:1000) were obtained from Cell Signaling Technology (Danvers, MA, USA). DOX, 5-FU, and VIN were purchased from Sigma-Aldrich (St. Louis, MO, USA).

### 2.2. Sample Preparation

Non-fermented noni extract (NFN) and FN were provided by NSTBIO (Incheon, Korea). Noni fruits were harvested from Java Island (Indonesia), washed, and squeezed to obtain the juice, which was then freeze-dried to produce NFN. For FN, noni fruits were fermented at 37 ± 2 °C for 60 d using seven species of lactic acid bacteria starters (including *Lactiplantibacillus plantarum*, *Lacticaseibacillus rhamnosus*, *Lacticaseibacillus casei*, *Limosilactobacillus fermentum*, *Lacticaseibacillus paracasei*, *Lactococcus lactis* subsp. *lactis*, and *Limosilactobacillus reuter*) at 2% (*w*/*w*), then squeezed, and the fermented juice was freeze-dried to produce FN. The analysis results for NFN (lot No. ON230531) and FN (lot No. NST230728001) used in this study are presented in Figures S1 and S2 and Table S1.

### 2.3. Cell Culture

A549 (CCL-185), MCF-7 (HTB-22), and SH-SY5Y (CRL-2266) cells were purchased from ATCC. A549 cells were cultured in F-12K medium supplemented with 10% FBS and 1% P/S. MCF-7 cells were cultured in EMEM supplemented with 10% FBS and 1% P/S. SH-SY5Y cells were cultured in EMEM:F-12 (1:1) supplemented with 10% FBS and 1% P/S. All cells were incubated at 37 °C in a humidified atmosphere with 5% CO_2_.

### 2.4. Cell Viability and Synergistic Effects of A549, MCF-7, and SH-SY5Y Cells with XTT Assays and Combination Index

A549, MCF-7, and SH-SY5Y cells were seeded in 96-well plates at a density of 1 × 10^4^ cells/well and incubated for 24 h, to allow cell adherence. A549 cells were treated with 5-FU (12.5 to 200 μM), NFN (200 to 3200 μg/mL), FN (200 to 3200 μg/mL), and a combination of 5-FU and NFN or FN (5-FU/NFN or FN) at various concentration combinations (i.e., 12.5/800, 12.5/1600, 12.5/3200, 25/800, 25/1600, 25/3200, 50/800, 50/1600, and 50 μM/3200 μg/mL). MCF-7 cells were treated with DOX (3.125 to 50 μM), NFN (200 to 3200 μg/mL), FN (200 to 3200 μg/mL), and a combination of DOX and NFN or FN (DOX/NFN or FN) at various concentration combinations (i.e., 12.5/800, 12.5/1600, 12.5/3200, 25/800, 25/1600, 25/3200, 50/800, 50/1600, and 50 μM/3200 μg/mL). SH-SY5Y cells were treated with VIN (0.001 to 10 μM), NFN (200 to 3200 μg/mL), FN (200 to 3200 μg/mL), and a combination of VIN and NFN or FN (VIN/NFN or FN) at various concentration combinations (i.e., 0.001/800, 0.001/1600, 0.001/3200, 0.01/800, 0.01/1600, 0.01/3200, 0.1/800, 0.1/1600, and 0.1 μM/3200 μg/mL). Untreated cells (no drug and no NFN or FN treatment) were used as the control group for all experiments. After 24, 48, and 72 h of incubation, cell viability was assessed using the XTT assay, following standard procedures. The CompuSyn computer program (www.combosyn.com (accessed on 5 December 2024)) was used to quantify the synergism of 5-FU, DOX, VIN, and FN or NFN combinations using the Chou-Talalay method [17]. The synergism of 5-FU, DOX, VIN, and FN or NFN co-treatment was evaluated by calculating the combination index (CI) value, wherein CI < 0.5 indicated strong synergism, 0.5< CI < 1.0 indicated moderate synergism, and 1.0 < CI indicated antagonism [18].

### 2.5. Cell Migration

Migration of A549, MCF-7, and SH-SY5Y cells was assessed using the scratch wound assay. These cells were seeded in 6-well plates at a density of 1 × 10^6^ cells/well and incubated for 24 h, to allow cell adherence. The cell monolayer was carefully scratched with a yellow pipette tip and washed with PBS to remove cell debris. A fresh medium containing 5-FU (50 µM), DOX (50 µM), VIN (0.1 µM), and FN (3200 µg/mL) alone or in combination was added to the corresponding wells for 24 h. At 0 and 24 h, scratch wounds in each well were photographed using a microscope (CKX41; Olympus, Tokyo, Japan). Cell migration was quantified using the ImageJ software (ver. 1.53e, NIH, Bethesda, MD, USA).

### 2.6. Colony Formation

A modified method was used to assess early colony formation [19]. A549, MCF-7, and SH-SY5Y cells were seeded in 6-well plates at a density of 1 × 10^3^ cells/well and incubated for 24 h to allow cell attachment. A549 cells were treated with 5-FU (50 µM), FN (3200 µg/mL), and 5-FU (50 µM) combined with FN (3200 µg/mL). MCF-7 cells were treated with DOX (50 µM), FN (3200 µg/mL), and DOX (50 µM) combined with FN (3200 µg/mL); SH-SY5Y cells were treated with VIN (0.1 µM), FN (3200 µg/mL), and VIN (0.1 µM) combined with FN (3200 µg/mL). Every 3 d, the complete culture medium containing the anticancer drugs, FN, or a combination of anticancer drugs and FN was replaced, and the cells were cultured for 10 d. After incubation, the cells were washed thrice with PBS, fixed with 4% formaldehyde for 15 min, and stained with 5% crystal violet for 30 min. The stained cells were air-dried, and the number of colonies was quantified using the ImageJ software.

### 2.7. Analysis of Apoptosis-Related Protein Expression Using Western Blotting

A549 cells were pre-treated with 5-FU (50 μM), FN (3200 μg/mL), and 5-FU (50 μM) combined with FN (3200 μg/mL) for 24 h; MCF-7 cells were pre-treated with DOX (50 μM), FN (3200 μg/mL), and DOX (50 μM) combined with FN (3200 μg/mL) for 24 h; and SH-SY5Y cells were pre-treated with VIN (0.1 μM), FN (3200 μg/mL), and VIN (0.1 μM) combined with FN (3200 μg/mL) for 24 h. The cells in each group were washed thrice with PBS and lysed in lysis buffer. After centrifugation at 12,000× *g* at 4 °C for 15 min, proteins in the supernatant were quantified using a Bradford protein assay kit. Proteins were separated using 10% sodium dodecyl sulfate–polyacrylamide gel electrophoresis and transferred to a polyvinylidene fluoride membrane. Membranes were blocked in tris-buffered saline with tween 20 (TBST) solution containing 5% bovine serum albumin or nonfat milk for 1 h. The primary antibody was incubated at 4 °C overnight, and the secondary antibodies were incubated at 23 °C for 1 h. Western blotting bands were observed using the ChemiDoc image software (version 5.2.1, Bio-Rad, Hercules, CA, USA).

### 2.8. Statistical Analyses

The results are expressed as mean ± standard deviation. All statistical analyses were performed using the SPSS software (version 24.0; SPSS, Chicago, IL, USA). Statistical significance was determined using the independent sample *t*-test. Differences were considered statistically significant at * *p* < 0.05, ** *p* < 0.01, and *** *p* < 0.001 vs. control and at # *p* < 0.05, ## *p* < 0.01, and ### *p* < 0.001 vs. single-drug treatment. Spearman’s rank correlation analysis was conducted to evaluate the relationship among the expression levels of apoptosis-related proteins. The resulting correlation coefficients (r) and *p*-values were visualized using a correlation heatmap.

## 3. Results

### 3.1. Cell Viability and Synergistic Effects of A549, MCF-7, and SH-SY5Y Cells with XTT Assays and Combination Index

#### 3.1.1. Anticancer Effects of 5-FU, DOX, VIN, NFN, and FN in A549, MCF-7, and SH-SY5Y Cells

**Figure 1:**
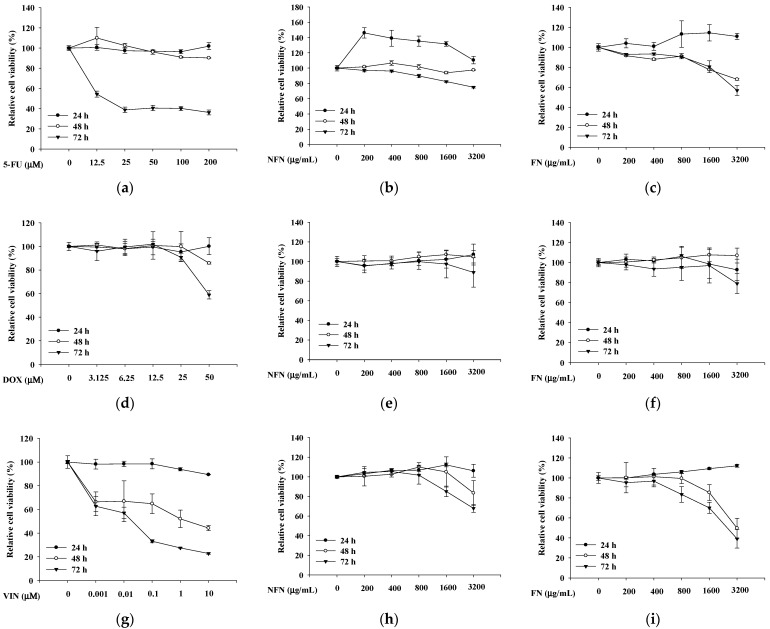
The inhibition of cell viability by 5-FU, DOX, VIN, NFN, and FN in A549, MCF-7, and SH-SY5Y cell lines. (**a**–**c**) Inhibitory effects of 5-FU (**a**), NFN (**b**), and FN (**c**) on A549 cell viability. (**d**–**f**) Inhibitory effects of DOX (**d**), NFN (**e**), and FN (**f**) on MCF-7 cell viability. (**g**–**i**) Inhibitory effects of VIN (**g**), NFN (**h**), and FN (**i**) on SH-SY5Y cell viability. Data are expressed as mean ± standard deviation (SD) (n = 3). 5-FU: 5-fluorouracil; DOX: doxorubicin; VIN: vincristine; NFN: non-fermented noni extract; FN: fermented noni extract.

After 72 h of treatment, the anticancer effects of 5-FU (Figure 1a), NFN (Figure 1b), and FN (Figure 1c) were evaluated using the XTT assay. In A549 cells, 5-FU, NFN, and FN significantly reduced cell viability in a dose-dependent manner. Similarly, DOX (Figure 1d), NFN (Figure 1e), and FN (Figure 1f) decreased MCF-7 cell viability in a dose-dependent manner. In SH-SY5Y cells, VIN (Figure 1g), NFN (Figure 1h), and FN (Figure 1i) exhibited dose-dependent anticancer effects.

#### 3.1.2. Synergistic Anticancer Effects of 5-FU, DOX, and VIN Combined with NFN or FN in A549, MCF-7, and SH-SY5Y Cells

**Figure 2:**
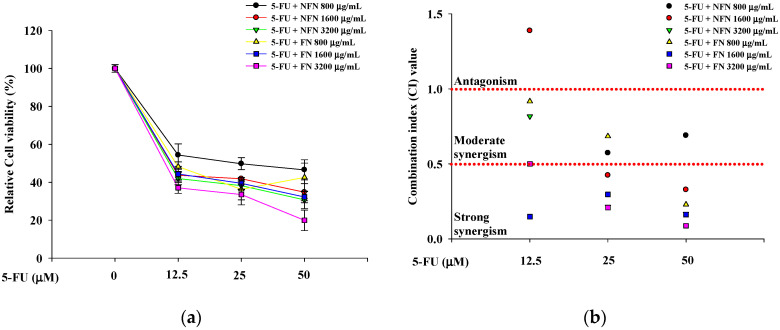

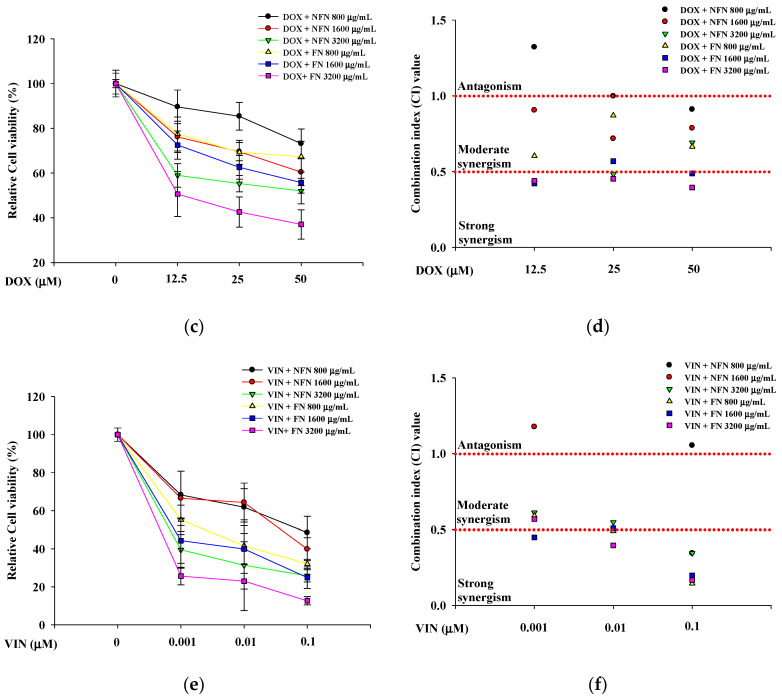
Enhanced anticancer effects of 5-FU, DOX, and VIN in combination with NFN or FN on cancer cells after 72 h of treatment. (**a**) Relative cell viability of A549 cells treated with 5-FU combined with NFN or FN at different concentrations. (**b**) Combination index (CI) values of different combinations were analyzed in A549 cells. (**c**) Relative cell viability of MCF-7 cells treated with DOX combined with NFN or FN at different concentrations. (**d**) CI values of different combinations were analyzed in MCF-7 cells. (**e**) Relative cell viability of SH-SY5Y cells treated with VIN in combination with NFN or FN at different concentrations. (**f**) CI values of different combination tests were analyzed in SH-SY5Y cells. The dashed horizontal line at CI = 1.0 indicates the threshold between antagonism (>1.0) and synergism (<1.0). All experiments were performed using the XTT assay. Data are expressed as mean ± SD (n = 3). 5-FU: 5-fluorouracil; DOX: doxorubicin; VIN: vincristine; NFN: non-fermented noni extract; FN: fermented noni extract.

When evaluating drug combinations, synergistic effects are typically quantified based on the drug CI [20]. To evaluate whether the combination of anticancer agents with NFN or FN exhibited synergistic anticancer effects, cell viability was assessed in A549, MCF-7, and SH-SY5Y cells treated with various concentration combinations (Figure 2). In A549 cells, three concentrations of 5-FU (12.5, 25, and 50 μM) and three concentrations of NFN or FN (800, 1600, and 3200 μg/mL) were paired to form nine different combinations (5-FU/NFN or FN: 12.5/800, 12.5/1600, 12.5/3200, 25/800, 25/1600, 25/3200, 50/800, 50/1600, and 50 μM/3200 μg/mL) (Figure 2a). FN consistently exhibited lower CI values than those of NFN, with the lowest CI value of 0.087 observed for the combination of 50 μM 5-FU and 3200 μg/mL FN (Figure 2b). Similarly, in MCF-7 cells, three concentrations of DOX (12.5, 25, and 50 μM) and three concentrations of NFN or FN (800, 1600, and 3200 μg/mL) were paired to generate nine combinations (DOX/NFN or FN: 12.5/800, 12.5/1600, 12.5/3200, 25/800, 25/1600, 25/3200, 50/800, 50/1600, and 50 μM/3200 μg/mL) (Figure 2c). Correspondingly, FN exhibited lower CI values than those of NFN, with the lowest CI value of 0.395 observed for the combination of 50 μM DOX and 3200 μg/mL FN (Figure 2d). In SH-SY5Y cells, three concentrations of VIN (0.001, 0.01, and 0.1 μM) and three concentrations of NFN or FN (800, 1600, and 3200 μg/mL) were paired to form nine different combinations (VIN/NFN or FN: 0.001/800, 0.001/1600, 0.001/3200, 0.01/800, 0.01/1600, 0.01/3200, 0.1/800, 0.1/1600, and 0.1 μM/3200 μg/mL) (Figure 2e). Similarly to the other two cell lines, FN treatment resulted in lower CI values than those with NFN treatment. Interestingly, the lowest CI value was observed with the combination of 0.1 μM VIN and 800 μg/mL FN, although the combination of 0.1 μM VIN and 3200 μg/mL FN also showed a strong synergistic effect, with a CI value of 0.167 (Figure 2f). Based on these results, 50 μM 5-FU and 3200 μg/mL FN for A549 cells, 50 μM DOX and 3200 μg/mL FN for MCF-7 cells, and 0.1 μM VIN and 3200 μg/mL FN for SH-SY5Y cells.

### 3.2. Effects of 5-FU, DOX, and VIN Combined with NFN or FN on Cell Migration in A549, MCF-7, and SH-SY5Y Cells

**Figure 3:**
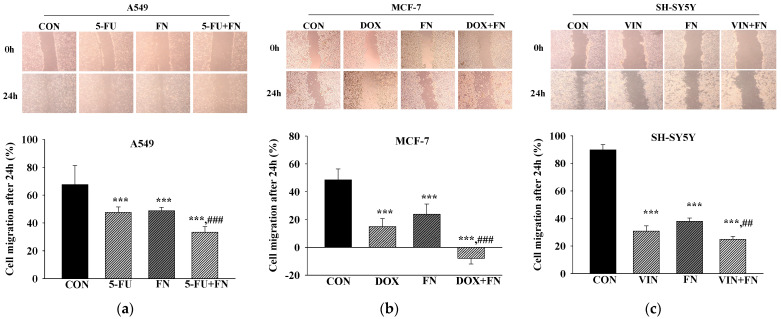
Combined treatment with 5-FU, DOX, or VIN and FN reduces the migration of A549, MCF-7, and SH-SY5Y cells in the scratch wound assay. Representative images and quantitative analysis of (**a**) A549, (**b**) MCF-7, and (**c**) SH-SY5Y cell migration following treatment with 5-FU, DOX, VIN, FN, or their respective combinations. Data are expressed as mean ± SD (n = 3). 5-FU: 5-fluorouracil (50 μM); DOX: doxorubicin (50 μM); VIN: vincristine (0.1 μM); FN: fermented noni extract (3200 μg/mL); 5-FU + FN: 5-FU (50 μM) + FN (3200 μg/mL); DOX + FN: DOX (50 μM) + FN (3200 μg/mL); VIN + FN: VIN (0.1 μM) + FN (3200 μg/mL). *** *p* < 0.001 vs. control; ## *p* < 0.01, ### *p* < 0.001 vs. single-drug treatment.

The effects of combined treatment with the anticancer drugs and FN 3200 μg/mL on cell migration in A549, MCF-7, and SH-SY5Y cells were evaluated using the scratch wound assay (Figure 3). Single treatment with either FN or each anticancer drug significantly inhibited cell migration, and the combined treatment further enhanced this inhibition. Thus, the combination of FN and anticancer drugs exerts a synergistic effect by suppressing cell migration in A549, MCF-7, and SH-SY5Y cells.

### 3.3. Effects of 5-FU, DOX, and VIN Combined with NFN or FN on Colony Formation in A549, MCF-7, and SH-SY5Y Cells

**Figure 4:**
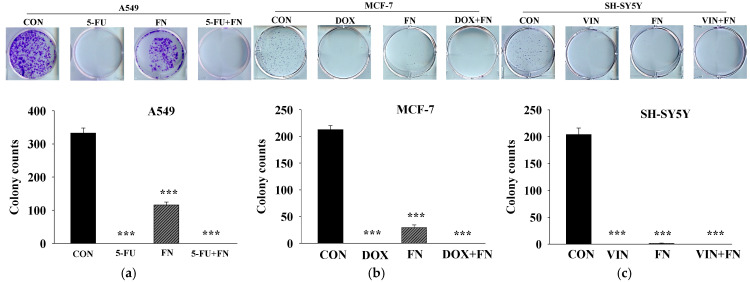
Combined treatment with 5-FU, DOX, or VIN and FN inhibits colony formation of A549, MCF-7, and SH-SY5Y cells. Representative images and quantitative analysis from colony formation assays conducted on (**a**) A549, (**b**) MCF-7, and (**c**) SH-SY5Y cells treated with 5-FU, DOX, VIN, FN, or their respective combinations. Data are expressed as mean ± SD (n = 3). 5-FU: 5-fluorouracil (50 μM); DOX: doxorubicin (50 μM); VIN: vincristine (0.1 μM); FN: fermented noni extract (3200 μg/mL); 5-FU + FN: 5-FU (50 μM) + FN (3200 μg/mL); DOX + FN: DOX (50 μM) + FN (3200 μg/mL); VIN + FN: VIN (0.1 μM) + FN (3200 μg/mL). *** *p* < 0.001 vs. control.

Effects of combined treatment with the anticancer drugs and FN 3200 μg/mL on colony formation in A549, MCF-7, and SH-SY5Y cells were evaluated (Figure 4). FN alone significantly inhibited colony formation, although the degree of inhibition varied among the cell lines, potentially due to differences in proliferation rates [21]. No colonies were observed in cells treated with each anticancer drug alone or the combination treatment. Although the synergistic effect on colony formation could not be quantitatively assessed in this study, imaging results demonstrated that FN alone inhibited cancer cell proliferation.

### 3.4. Effects of 5-FU, DOX, and VIN Combined with NFN or FN on Apoptosis-Related Signaling Pathways in A549, MCF-7, and SH-SY5Y Cells

**Figure 5:**
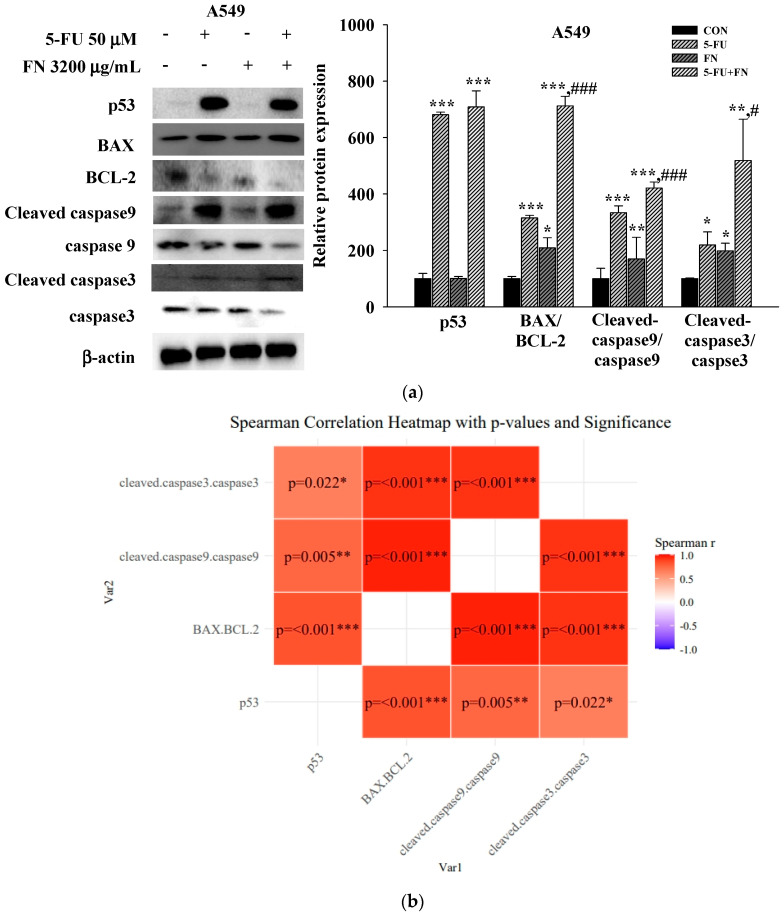

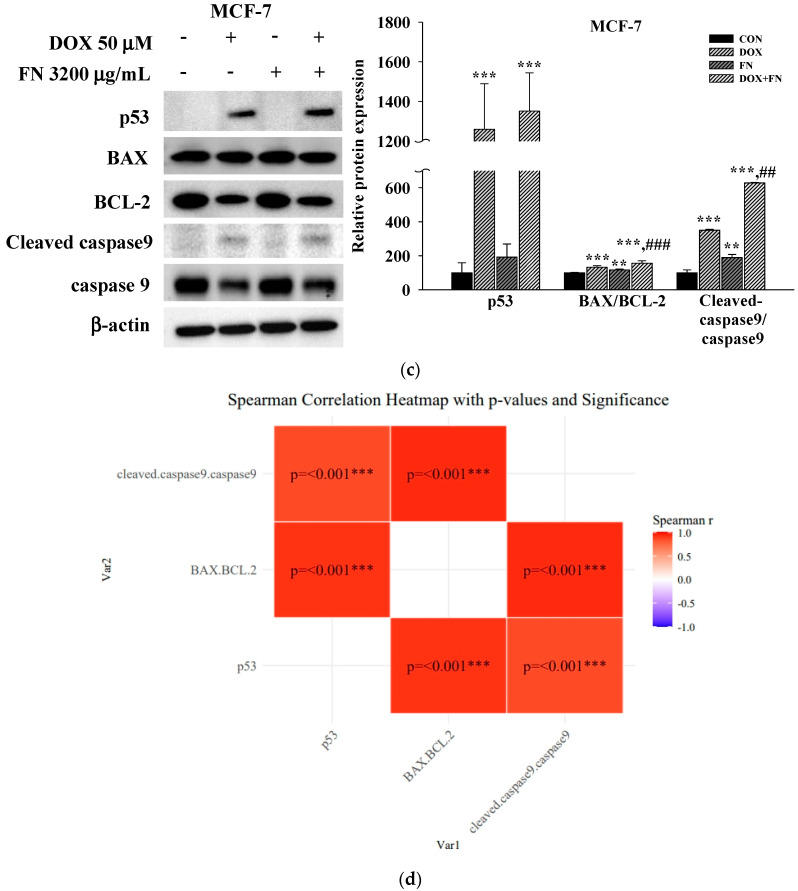

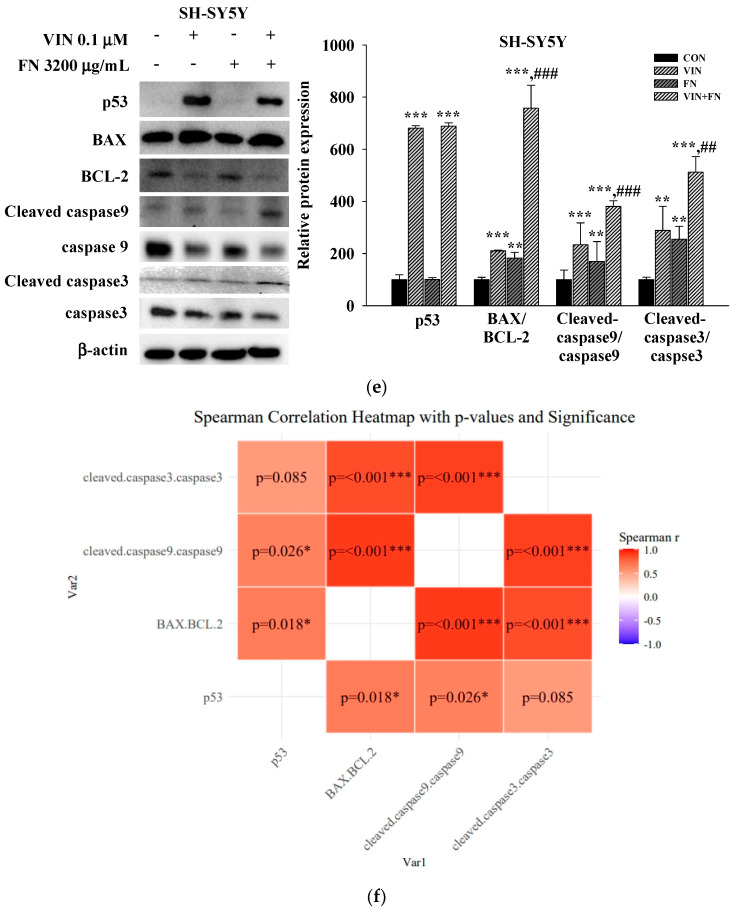
Combined treatment with 5-FU, DOX, or VIN and FN modulates apoptosis-related protein expression in A549, MCF-7, and SH-SY5Y cells. (**a**) Western blot analysis and (**b**) Spearman correlation analysis of p53, BAX, BCL-2, cleaved caspase-9, caspase-9, cleaved caspase-3, and caspase-3 in A549 cells; (**c**) Western blot analysis and (**d**) Spearman correlation analysis of p53, BAX, BCL-2, cleaved caspase-9, and caspase-9 in MCF-7 cells; (**e**) Western blotting and (**f**) Spearman correlation analysis of p53, BAX, BCL-2, cleaved caspase-9, caspase-9, cleaved caspase-3, and caspase-3 in SH-SY5Y cells after treatment with 5-FU, DOX, VIN, FN or their respective combination for 24h. Densitometric quantification of Western blotting bands was performed using ImageJ and normalized to β-actin levels. The results are presented as a percentage of the control group. Data are expressed as mean ± SD (n = 3). 5-FU: 5-fluorouracil (50 μM); DOX: doxorubicin (50 μM); VIN: vincristine (0.1 μM); FN: fermented noni extract (3200 μg/mL); 5-FU + FN: 5-FU (50 μM) + FN (3200 μg/mL); DOX + FN: DOX (50 μM) + FN (3200 μg/mL); VIN + FN: VIN (0.1 μM) + FN (3200 μg/mL). * *p* < 0.05, ** *p* < 0.01, *** *p* < 0.001 vs. control; # *p* < 0.05, ## *p* < 0.01, ### *p* < 0.001 vs. single-drug treatment.

To investigate the mechanisms underlying the observed synergistic and pro-apoptotic effects of combining anticancer drugs (50 μM 5-FU, 50 μM DOX, and 0.1 μM VIN) with FN (3200 μg/mL) in A549, MCF-7, and SH-SY5Y cells, the expression levels of key apoptosis-related proteins were assessed using Western blotting. Compared to treatment with each agent alone, 24 h of co-treatment with FN and either 5-FU or VIN in A549 (Figure 5a) and SH-SY5Y (Figure 5c) cells significantly increased the expression levels of intrinsic apoptosis-related proteins, including the BAX/BCL-2, cleaved caspase-9/caspase-9, and cleaved caspase-3/caspase-3 ratios. Interestingly, the expression of both cleaved caspase-3 and caspase-3 was undetectable in MCF-7 cells under all experimental conditions (Figure 5b). This result is consistent with the known caspase-3 deficiency in this cell line [22]. The expression pattern of other proteins was similar to that observed in the other cell lines. To further explore the relationship among these proteins, Spearman’s rank correlation analysis was performed, revealing strong positive correlations between p53 expression, BAX/BCL-2, cleaved caspase-9/caspase-9, and cleaved caspase-3/caspase-3 ratios across all cell lines (Figure 5b,d,f). These results indicate that increased expression of these proteins is interrelated under combination treatment.

## 4. Discussion

The anticancer potential of noni is well-established [23]. Seven major compounds, including asperulosidic acid, deacetylasperulosidic acid, scopoletin, morindoline, and three fatty acid glycosides, have been identified in noni juice [24]. Asperulosidic acid exerts anticancer effects by inhibiting AP-1 transcriptional activation and cell transformation in mouse epithelial JB6 cells [25]. Scopoletin exerts anticancer effects, such as apoptosis, cell cycle arrest, and inhibition of cell invasion, by inducing the PI3K/AKT signaling pathway in human cervical cancer cell lines [26]. Rutin has also demonstrated anticancer effects in hepatoma cell lines [27]. Additionally, trace compounds such as quercetin, kaempferol, and chrysin in noni extracts exhibit anticancer activities [9,28]. However, to our knowledge, the anticancer activity of deacetylasperulosidic acid has not yet been reported. In the present study, we investigated the potential of FN as a synergistic adjuvant to conventional chemotherapy by assessing its combined cytotoxic and pro-apoptotic effects with 5-FU, DOX, and VIN in various cancer cell lines (A549, MCF-7, and SH-SY5Y). Our results demonstrated that both NFN and FN induced intrinsic apoptosis and reduced cell viability in a dose-dependent manner. Overall, FN exhibited stronger anticancer activity than that of NFN in all tested cell models. Chemical analysis revealed that FN contained lower levels of asperulosidic acid but higher levels of deacetylasperulosidic acid than those in NFN (Table S1), suggesting that fermentation promoted the hydrolysis of glycosidic bonds and conversion of iridoid glycosides into more bioavailable forms [29,30]. Importantly, when combined with chemotherapeutic agents, FN further enhanced cytotoxicity beyond the effects observed with anticancer drugs alone, suggesting a synergistic interaction.

To further elucidate the mechanisms underlying these enhanced anticancer effects, we performed Western blot analysis of key apoptosis-related proteins. Treatment with FN alone did not significantly affect p53 expression but significantly increased the expression ratios of BAX/BCL-2, cleaved caspase-9/caspase-9, and cleaved caspase-3/caspase-3 in A549 and SH-SY5Y cells. In contrast, treatment with 5-FU, DOX, or VIN alone significantly upregulated p53 and increased BAX/BCL-2 and caspase cleavage. MCF-7 cells exhibited the same trend as A549 and SH-SY5Y cells, except that cleaved caspase-3 and caspase-3 were not expressed. Correlation heatmaps revealed significant positive correlations between p53 expression, BAX/BCL-2 ratio, and cleaved caspase-9/caspase-9 ratio across all three cell lines. These results are consistent with those of previous studies reporting that noni components promote apoptosis through p53-dependent mechanisms. Damnacanthal, a major phytochemical in noni, induces apoptosis by upregulating p53 and p21 expression, along with activating caspase-7 in breast cancer cells [31,32]. Noni extract also enhances p53 and Bax expression in cervical cancer cells [33]. Moreover, xeronine contributes to anticancer activity by modifying misfolded proteins and inhibiting COX-2-mediated angiogenesis [34]. These findings support the notion that the pro-apoptotic effects of FN observed in our study may be attributed to the synergistic actions of multiple bioactive compounds targeting apoptotic signaling pathways.

The observed synergistic effects between FN and anticancer drugs are of particular significance. Chemoresistance often arises due to impaired apoptotic signaling, and agents that restore or enhance apoptosis may improve therapeutic outcomes. Our findings that FN combined with anticancer drugs further upregulated the BAX/BCL-2 and cleaved caspase-9/caspase-9 ratios relative to drug treatment alone support the hypothesis that FN sensitizes cancer cells to apoptosis. Despite these promising results, this study has several limitations. All experiments were performed in vitro; therefore, the synergistic effects of FN observed in cancer cell lines require in vivo validation to assess pharmacokinetics, bioavailability, dose distribution, and systemic toxicity under physiological conditions. The mechanistic analysis focused only on intrinsic apoptosis markers, even though FN may also regulate upstream signaling events such as ROS generation, mitochondrial membrane potential disruption, or PI3K/AKT modulation, which were not examined in this study [25,27]. Differences in chemical composition between NFN and FN were confirmed, but the specific bioactive metabolites responsible for the enhanced synergistic effects remain unidentified, and their characterization will require metabolomic, fractionation, or target-based analyses. Only one optimized concentration combination was evaluated for each chemotherapeutic agent, and the clinical relevance of the in vitro doses has yet to be established. This study also did not examine the impact of FN in drug-resistant cancer cell models, even though resistance to chemotherapeutic agents such as doxorubicin frequently limits treatment efficacy. In future studies, we plan to perform in vivo efficacy experiments, conduct broader mechanistic analyses, identify key active constituents, and evaluate FN in chemoresistant cancer cell lines to further clarify its therapeutic potential in combination chemotherapy.

## 5. Conclusions

**Figure 6:**
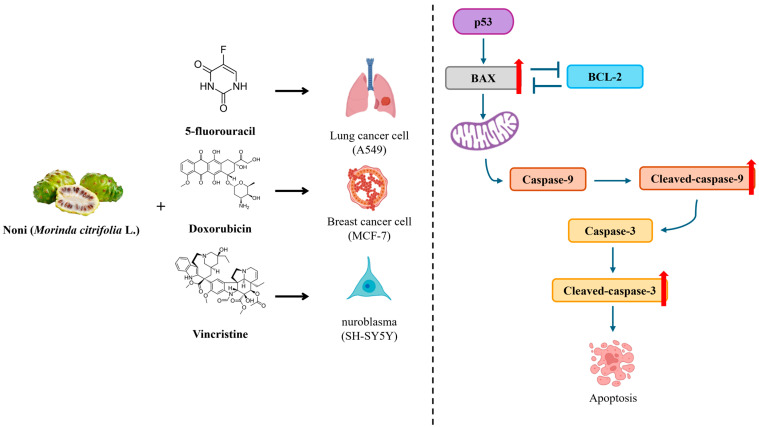
Schematic summary of the synergistic anticancer effect mechanism of fermented noni extract combined with 5-fluorouracil, doxorubicin, or vincristine via the intrinsic apoptotic pathway. The combination treatment enhances p53-mediated BAX activation, inhibits BCL-2, and promotes caspase-9 and caspase-3 cleavage, ultimately inducing apoptosis in A549, MCF-7, and SH-SY5Y cells.

Our study demonstrated that FN enhances the anticancer efficacy of 5-FU, DOX, and VIN by promoting apoptosis via the intrinsic pathway (Figure 6). Thus, FN may serve as a promising adjuvant to potentiate the therapeutic effects of conventional chemotherapy. However, this study has several limitations. First, all experiments were performed in vitro; therefore, the synergistic effects observed in cancer cell lines require validation in in vivo models to assess pharmacokinetics, bioavailability, and systemic toxicity. Second, the mechanistic analysis focused solely on intrinsic apoptosis markers, while other pathways—including ROS generation, PI3K/AKT signaling, or autophagy—may also contribute to the synergistic effects of FN. Third, although differences in chemical composition between NFN and FN were confirmed, the specific bioactive metabolites responsible for the enhanced anticancer activity remain unidentified, warranting further metabolomic or fractionation studies. Future investigations should include in vivo validation, broader mechanistic analyses, and identification of key active constituents to fully elucidate the therapeutic potential and clinical applicability of FN.

## Data Availability

The original contributions presented in this study are included in the article/Supplementary Material. Further inquiries can be directed to the corresponding author(s).

## Supplementary Materials

The following supporting information can be downloaded at: https://www.mdpi.com/article/10.3390/cimb47120993/s1, Figure S1: Certificate of analysis for NFN used in this study; Figure S2: Certificate of analysis for FN used in this study; Table S1: Chemical composition of non-fermented noni extract (NFN) and fermented noni extract (FN).
