# Supplementary material for: Synergistic Anticancer Effects of Fermented Noni Extract Combined with 5-Fluorouracil, Doxorubicin, and Vincristine on A549, MCF-7, and SH-SY5Y Cell Models
Source: Curr Issues Mol Biol. 2025 Nov 27;47(12):993. doi: 10.3390/cimb47120993 (PMC12731836; doi:10.3390/cimb47120993)
Supplement: Supplementary file 1 [file cimb-47-00993-s001.zip › Supplementary material Figure S2.pdf]

NSTBIO Co.,Ltd.
102, Goeumdal-ro, Yangchon-eup, Gimpo-si,
Gyeonggi-do, Republic of Korea
Tel +82-31-996-5123 Fax +82-31-996-5124
www.nstbio.co.kr

NSTBIO

Certificate of Analysis

Product Name : Fermented noni extract

LOT No. : NST230728001

Manufacturing date : 2024.06.07

Analytical Tests	Results
Appearance	dark brown coloured powder with characteristic odour
Water content (%, w/w)	1.90
Asperulosidic acid (mg/g)	1.19
Deacetylasperulosidic acid (mg/g)	15.93
Scopoletin (mg/g)	0.43

Inspector : Ji Soo, Choi

Date : 2024. 06. 13

NSTBIO CO., Ltd. R&D Center

Tel. +82-32-715-5912 / Fax. +82-32-715-5913

Figure S2. Certificate of analysis for FN used in this study.
