# Supplementary material for: Synergistic Anticancer Effects of Fermented Noni Extract Combined with 5-Fluorouracil, Doxorubicin, and Vincristine on A549, MCF-7, and SH-SY5Y Cell Models
Source: Curr Issues Mol Biol. 2025 Nov 27;47(12):993. doi: 10.3390/cimb47120993 (PMC12731836; doi:10.3390/cimb47120993)
Supplement: Supplementary file 1 [file cimb-47-00993-s001.zip › Supplementary material Table S1.pdf]

Supplymental material

Table S1. Chemical composition of non fermented noni extract (NFN) and fermented noni extract (FN)

Analytical test	NFN	FN
Lot No.	ON230531	NST230728001
Asperulosidic acid (mg/g)	9.47	1.19
Deacetylasperulosidic acid (mg/g)	12.93	15.93
Scopoletin (mg/g)	0.45	0.43
